# Quantification of left atrial flow velocity distribution in atrial fibrillation using 4D flow MRI

**DOI:** 10.1186/1532-429X-15-S1-P261

**Published:** 2013-01-30

**Authors:** Jacob U Fluckiger, Jeffrey J Goldberger, Daniel C Lee, Jason Ng, Richard Lee, Andrew B Olsen, James Carr, Michael Markl

**Affiliations:** 1 Northwestern University, Chicago, IL, USA; 2 Northwestern Memorial Hospital, Chicago, IL, USA

## Background

Atrial fibrillation (AF) is an arrhythmia characterized by irregular electrical activity in the left atrium (LA). Thromboembolism is the most serious complication of AF, usually manifesting as stroke or systemic embolism (1). This is thought to be linked to the increased risk of thrombus formation in the LA due to a decrease in blood velocity (stasis) or flow abnormalities. A better appreciation of the underlying mechanisms and risk factors for thrombus formation in the individual patient are needed to improve risk stratification and therapy planning. Using 4D-flow MRI, time resolved blood velocity measurements can be made in patients with AF. The aim of this study was to compare LA velocity distributions in different groups of AF patients. We hypothesize that persistent AF results in more deranged LA flow patterns with reduced velocities which may be related to an increased risk of thrombus formation and thus stroke.

## Methods

**Figure 1:**
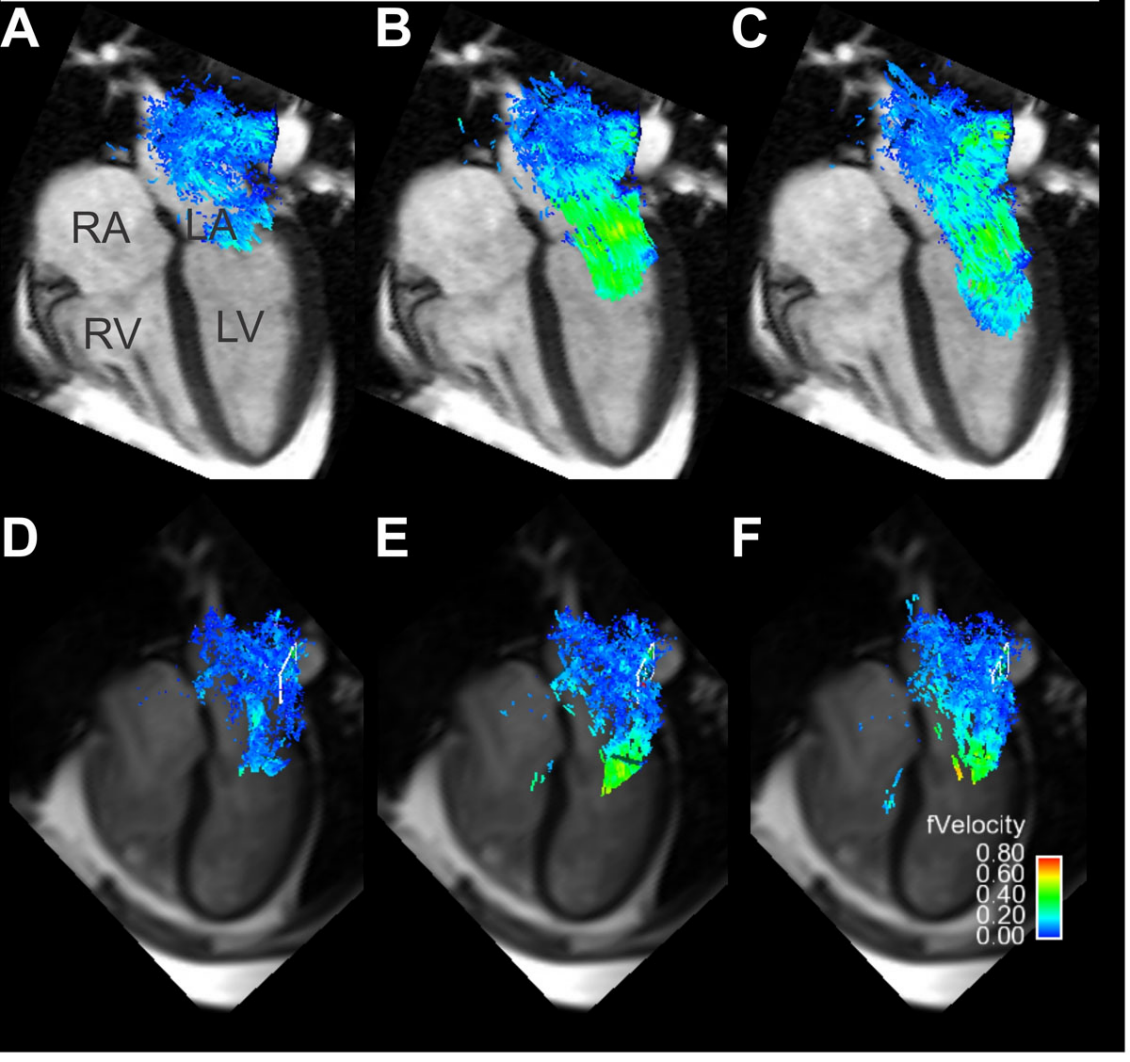
A three-dimensional representation of left atrial blood flow in two patients with atrial fibrillation. Patient 1 (A-C) was in sinus rhythm at the time of imaging, and exhibits more regular flow patterns throughout the cardiac cycle. Patient 2 (D-F) was in persistent AF at the time of imaging and displays a more disorderly flow pattern as well as lower overall velocities.

MRI data were acquired from 31 AF patients (mean age 63+/-10.5) from two groups (n=21 post-treatment, in sinus rhythm, n=10 in persistent AF). Each subject underwent gated 4D-Flow MRI on 1.5T and 3T MR systems (Siemens, Erlangen, Germany). After noise filtering, Maxwell, and eddy current correction, time resolved pathlines were emitted from planes placed manually over the pulmonary veins at the junction with the LA (Fig 1). The LA was segmented and the distribution of velocities was quantified by histogram analysis. Mean velocities were calculated for each subject group. In addition number of voxels with velocities greater than 0.2m/s was calculated. The selection of the velocity threshold was based on previous TEE results which found that systolic LA velocities less than 0.2m/s constitute a risk factor for stroke (2).

## Results

**Figure 2:**
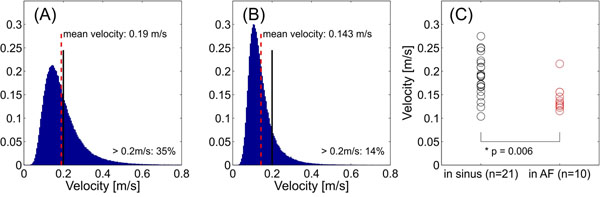
Mean LA velocity histograms for 21 patients in sinus rhythm (A) and 10 patients in AF (B) at the time of imaging. The mean velocity and the percent of velocity values greater than 0.2 m/s are marked each panel. The area of each histogram is normalized to unity. Panel (C) displays mean velocities from the top tertile of blood flow within the left atrium for the groups in panels (A-B). A signfican reduction (i.e. increased stasis) for persistant AF is evident.

Qualitative observation of the blood flow patterns in patients showed very different patterns between those subjects imaged in sinus rhythm and those in persistent AF. Fig 1 displays clear differences in blood flow at end systole (A,D), early diastole (B,E), and mid diastole (C,F) for a post-intervention AF patient in sinus rhythm and in persistent AF. The quantitative results from all subjects are summarized in fig 2. Patients with persistent AF had a mean blood flow of 0.14±0.03m/s, lower than AF patients in sinus rhythm (0.19±0.04m/s, p=0.006). 14% of the velocity values for patients in AF were greater than 0.2m/s, lower than patients in sinus (35%, p=0.004).

## Conclusions

Using 4D flow MRI we showed that patients with persistent AF have significantly lower LA blood velocity than patients in sinus rhythm. Future work will focus on improving the 4D flow acquisition for regions of low or incoherent flow, using 4D flow to monitor the treatment of patients with AF, and assessing whether flow quantification metrics can serve as biomarkers for stratifying risk of stroke and other AF complications. 1 Wolf et al., Stroke 22. 2 Bernhardt et al. JACC 45(11).

## Funding

AHA 12GRNT12080032

